# Obituary

**Published:** 1879-12

**Authors:** 


### Obituary.

IN Ashton, on Tuesday, October 28th, of heart disease, D. H. Spickler, M.D., aged 49 years.

Dr. Spickler was born in Middleburg, Franklin county, Pa., March 18, 1830, and came west sometime during the year 1853. He was graduated in his profession at Rush Medical College, Chicago, in 1857, soon after which he entered into partnership with Dr. Hewitt, of Franklin Grove, in the practice of medicine. The partnership was eminently successful and continued until sometime during the year 1869, when he entered into relations with P. C. Rooney, in connection with a drug store. In 1871 he left the drug business and became editor and proprietor of the Franklin Grove *Reporter*, which he conducted with much energy and ability for four years. Soon after disposing of the *Reporter* he purchased the Mendota *News*, which flourished under his able management for two years, when he moved to Ashton and again entered the practice of medicine.

The doctor was a man of culture and of rare abilities. Possessing the finest memory, combined with energy, application and a love for books; he became a perfect storehouse of knowledge, and upon all subjects conversed with ease and freedom in his own inimitable style. In the practice of his profession he kept pace with the times. His style as a writer was peculiarly his own. Terse, clear and pointed, he never left his readers in doubt as to his meaning. He stripped his subject of all superfluities, and expressed his thoughts with frankness and independence in a few concise, often humorous, sentences.

But intellect is not all of man; there is something else that enshrines his memory in the heart of his fellow man, and perpetuates his influence long after the portals of the grave have closed over all that is mortal. There are noble impulses that spring from the soul, and like streams of pure water, flow out to gladden all who come within the circle of their influence. The doctor was no stranger to these impulses, as his strict integrity in business and his well known generosity testified. He was the friend of the poor, and during the war attended free of charge the families of those who went to the front. He was also a man to be admired for his social qualities, and the news of his sudden death has thrilled every household and saddened every heart with the feeling that a friend has been lost.

---

AT a meeting of the senior class of the Chicago Medical College, held Nov. 15, 1879, the following resolutions were adopted in relation to the decease of Mr. Brown.

*Whereas*, We have heard of the sudden death of Mr. A. S. Brown, a member of the senior class of the Chicago Medical College; therefore,

*Resolved*, That we sincerely regret the early death of our esteemed classmate, and deeply sympathize with his friends and relatives in this sad affliction.

*Resolved*, That we recognize in the life of our departed classmate a character of manliness and integrity worthy of the highest regard.

*Resolved*, That his customary seat among us in the senior lecture-room be duly draped, in memory of his departure.

*Resolved*, That copies of these resolutions be forwarded to the friends of the deceased, and also to the CHICAGO MEDICAL JOURNAL AND EXAMINER, and *Vidette*, for publication.

*Committee for the class*, { J. E. BUMSTEAD, G. W. MASON, J. M. G. CARTER.

